# Evaluation of a new digital pediatric malnutrition risk screening tool for hospitalized children with congenital heart disease

**DOI:** 10.1186/s12887-023-03899-1

**Published:** 2023-03-18

**Authors:** Yajie Zhang, Lina Lu, Ling Yang, Weihui Yan, Qun Yu, Jinye Sheng, Xiaomeng Mao, Yi Feng, Qingya Tang, Wei Cai, Ying Wang

**Affiliations:** 1 Division of Pediatric Gastroenterology and Nutrition, Xinhua Hospital, School of Medicine, Shanghai Jiao Tong University, Shanghai, China; 2 Shanghai Institute for Pediatric Research, Shanghai, China; 3 Department of Pediatric Surgery, Xinhua Hospital, School of Medicine, Shanghai Jiao Tong University, Shanghai, China; 4 Shanghai Key Laboratory of Pediatric Gastroenterology and Nutrition, Shanghai, China; 5 Pediatric Heart Center, Xinhua Hospital, School of Medicine, Shanghai Jiao Tong University, Shanghai, China; 6 Department of Nursing, Xinhua Hospital, School of Medicine, Shanghai Jiao Tong University, Shanghai, China; 7 Department of Clinical Nutrition, Xinhua Hospital, School of Medicine, Shanghai Jiao Tong University, Shanghai, China

## Abstract

**Background:**

In a cohort of hospitalized children with congenital heart disease (CHD), a new digital pediatric malnutrition screening tool as a mobile application was validated, and its effectiveness and clinical value were determined as a prospective study.

**Methods and results:**

Children with CHD (*n* = 1125) were screened for malnutrition risk. The incidence of risk and the differences among various age groups and types of CHD were characterized. The optimal threshold for the tool to determine if there is a risk of malnutrition is score 2, while the Youden index was 79.1%, and the sensitivity and specificity were 91.2% and 87.9%, respectively. Based on such criterion, 351 children were at risk of malnutrition accounting for 31.20% of the total. Compared with the non-malnutritional risk group, the median age for the group at risk for malnutrition was younger (8.641 months [4.8, 23.1] vs. 31.589 months [12.4, 54.3], *P* < 0.01), and the length of stay was longer (12.000 [8.0, 17.0] vs. (8.420 [5.0, 12.0], *P* < 0.01]. There were significant differences in malnutrition risk among different age groups (χ^2^ = 144.933, *P* < 0.01), and children under one year of age exhibited the highest risk for malnutrition and more extended hospital stay (H = 78.085, *P* < 0.01). The risk of malnutrition among children with cyanotic CHD was higher than in those with non-cyanotic CHD (χ^2^ = 104.384, *P* < 0.01).

**Conclusions:**

The new digital pediatric malnutrition screening tool showed high sensitivity and specificity in children with CHD. The tool indicated that the malnutrition risk for young children and children with cyanotic or Bethesda moderate and complex CHD was higher, and the hospitalization time was longer than in the non-risk group. The tool provides a rational approach to targeted nutrition intervention and support and may improve clinical outcomes.

## Background

Congenital heart disease (CHD) is one of the most common developmental abnormalities in children, with 4–10 cases per 1000 live births worldwide [1]. In China, CHD is the most common congenital abnormaly [2, 3]. Although most children with CHD exhibit normal weight at birth, due to abnormal hemodynamics, an increase in cardiopulmonary oxygen consumption, a diminution in energy intake, an elevation in sympathetic nerve activity, and feeding difficulties, these children lose weight secondary, in part, to a lack of specific nutrients or insufficient calorie intake [4–6]. Previous studies revealed that 15–41% of children with CHD developed malnutrition and growth disorders [1].

In hospitalized children, malnutrition precipitated by disease constitutes one of the principal reasons for prolonging the course of disease and increasing hospitalization time [7, 8]. Malnutrition affects growth and development, clinical prognosis, postoperative recovery, and length of hospital stay [6, 9]. It also can decrease bodily functions, and increase disease-related complications and mortality [10, 11]. For children with CHD, good nutritional status during the perioperative period is important, and therefore, nutritional risk screening and assessment should be completed in a timely manner. Appropriate intervention can accelerate the treatment and rehabilitation of the disease and shorten the recovery period and hospitalization days, reduce complications, lower costs, and improve clinical outcomes [8, 11, 12].

The nutritional risk screening tools presently used include the nutrition status and growth risk screening tool (STRONGkids), pediatric malnutrition assessment and screening tool (STAMP), Yorkhill pediatric malnutrition score (PYMS), digital measurement malnutrition risk screening tool (PediSmart), and the pediatric nutrition screening tool (PNST) [7, 13, 14]. However, the reliability and performance of these tools are inconsistent [14–22]. In China and elsewhere, there is still no universal pediatric nutritional-risk screening tool. The application of nutritional s tatus screening tools in children with CHD thus warrants further study.

Herein, we tested an optimized pediatric malnutrition-risk screening tool in hospitalized children with CHD. By investigating the detection rate for nutritional risk and analyzing the correlation between nutritional risk and clinical results, we evaluated the tool’s feasibility, its effectiveness, and its clinical value.

## Methods

### Subjects

Children hospitalized in Xinhua Hospital affiliated to the School of Medicine of Shanghai Jiao Tong University between May 2018 and November 2021 were selected prospectively. Inclusion criteria were (1) age 1 month—18 years corrected gestational age; (2) diagnosis of congenital heart disease; (3) admitted to the Pediatric Heart Center of the hospital; (4) a hospital stay length > 3 days; and (5) screening for malnutrition risk within 48 h after admission. The protocol was approved by the ethics committee of Xinhua Hospital affiliated to the School of Medicine of Shanghai Jiao Tong University (China; Approval No. XHEC-D-2020–071).

### Clinical data collection and anthropometric measurements

Patient sex, age, primary diagnosis, weight, height (at over two years of age) and length (at under two years of age), length of hospital stay, and nutrition intervention were recorded upon hospital admission and at discharge by the nursing staff. Body weight and length were measured with an infant scale with an attached infantometer (Seca 376 electronic baby scale; Seca Ltd, Hamburg, Germany) in children < 2 years of age and with an electronic scale and a stadiometer (RGZ-120; Shanghai Dongfang scales Co. Ltd, Shanghai, China) in children ≥ 2 years of age. Height was accurate to 0.1 cm, and weight was accurate to 0.1 kg. BMI was calculated as body weight (kg)/[length or height (m)]^2^. Z-scores were derived based on the WHO Child Growth standard charts [23].

### Screening of malnutrition risk

The screening tool was consolidated into a mobile application in this study, which could be downloaded in the app store (China) as “*Er Ke Ying Yang Shai Cha*(Pediatric Nutritional Risk Screening)”. The nurses involved in the study were installed with this app in their phones, and the accounts were managed by the administrator account. The pediatric malnutrition risk screening part was developed by our research team [22], which consisted of three elements: disease risk, dietary intake, and anthropometric indicator. The score for disease risk was determined according to the primary diagnosis based on the ICD-10, while the anthropometric indicator Z refers to weight for height values and was automatically calculated in the app (see Table 1 for details). Nurses were in charge of the screening (input the basic information of the three elements) and trained prior to the project. The same screening questions were used at admission and discharge. The risk results were automatically sent to registered dietitians and physicians via the application for further assessment and treatment with caloric intake evaluation and other variables.

**Table 1:** Pediatric malnutrition assessment screening tool

Project	Content	Score
Disease risk	Patent ductus arteriosus; Atrial septal defect; Atrioventricular septal defect (AVSD); Ventricular septal defect and 18 other types	0
	Pulmonary atresia; Severe pulmonary hypertension; Anomalous pulmonary venous drainage; Tetralogy of Fallot and eight other categories	1
Dietary intake	Normal, no obvious change	0
	Less than or equal to 50% less than usual in the previous month	1
	Over the previous month, eating decreased by more than 50% compared with normal	2
Anthropometric indicator	− 1 < Z	0
	− 2 < Z < − 1; BMI reflects overweight	1
	− 3 < Z < − 2; BMI reached obesity level	2
	Z ≤ − 3	3
Total score		0–6

### Criteria for malnutrition

For assessment of pediatric malnutrition risk, the WHO Z-score method is often used as a reference [24, 25]. Height for age Z value (HFA), weight for age Z value (WFA), and weight for height Z value (WFH) are suitable for children 0–5 years of age; and the age-specific body-mass index Z value (BMIZ) is suitable for individuals 5–18 years of age. We designated WFA or BMI Z <  − 2 as low weight, HFA <  − 2 as growth retardation, WFH <  − 2 as stunting, WFA > 2 or BMIZ > 1 as overweight, and WFA > 3 or BMIZ > 2 as obesity. The children found to be low weight, growth retardation, stunting or overweight were rated as malnutrition, and Z <  − 3 or Z > 3 denoted severe malnutrition [26]. In addition to the WHO standard, the American Society for Parenteral and Enteral Nutrition (ASPEN) criteria for malnutrition in children was used as a reference standard for comparison [27].

### Statistical treatment

The SPSS 21.0 software package was used for statistical analysis. R version 3.5.3 software was used for receiver operating characteristic (ROC) curve drawing. Non-normally distributed data are expressed as median (M) and interquartile interval (P25, P75). The Mann–Whitney U test was used for two groups of independent samples, and the Kruskal–Wallis H test for multiple independent samples. Counting data are expressed as rate (%) and were analyzed with the Chi-squared test. The specificity, sensitivity, and cut-off value were calculated and judged using ROC curves. The Youden index and kappa value were used to determine the consistency of screening results. Binary logit regression analysis was used to investigate which characteristics were correlated. *P* < 0.05 was considered statistically significant.

## Results

### General information

The study cohort included 1125 children with congenital heart disease. There were 575 boys (51.11%) with a median age of 21.40 (6.80, 45.01) months. The median age for the 550 girls (48.89%) was 25.12 (7.65, 50.84) months. The median Z scores for WFH, WFA, HFA, and BMI did not differ significantly between boys and girls. Consequently, gender was not considered in the analysis.

According to WHO standards, there were 272 cases of malnutrition at admission, with an incidence rate of 24.18%. Of these, 85 cases were graded as severe malnutrition, accounting for 31.25%. The incidence rates of underweight, retardation, and stunting were 142 cases (12.62%), 146 cases (12.98%), and 99 cases (8.80%), respectively. Forty-eight cases were overweight and 13 were obese (see Table 2).

**Table 2:** Congenital heart disease patient demographics

Basic features	N (%)
Sex	
Male	575 (51.11)
Female	550 (48.89)
Age (years)	
0–1	398 (35.38)
1–3	320 (28.44)
3–5	203 (18.04)
≥ 5	204 (18.13)
WHO standard assessment	
Underweight	142 (12.62)
Retardation	146 (12.98)
Stunting	99 (8.80)
Overweight	53 (4.83)
Obesity	13 (1.27)
Diagnosis	
Cyanotic	193 (17.16)
Non-cyanotic	932 (82.84)
Bethesda classification of CHD	
Simple	535 (47.56)
Moderate	390 (34.67)
Complex	200 (17.78)

There were 193 cases (17.16%) of cyanosis and 932 cases (82.84%) without cyanosis. Based on Bethesda classification of congenital heart disease, there were 535 (47.56%) simple cases, 390 (34.67%) moderate cases, and 200 (17.78%) cases with high severity.

### Tool validation and results

#### Validation of cut-off value for screening score

**Fig. 1:**
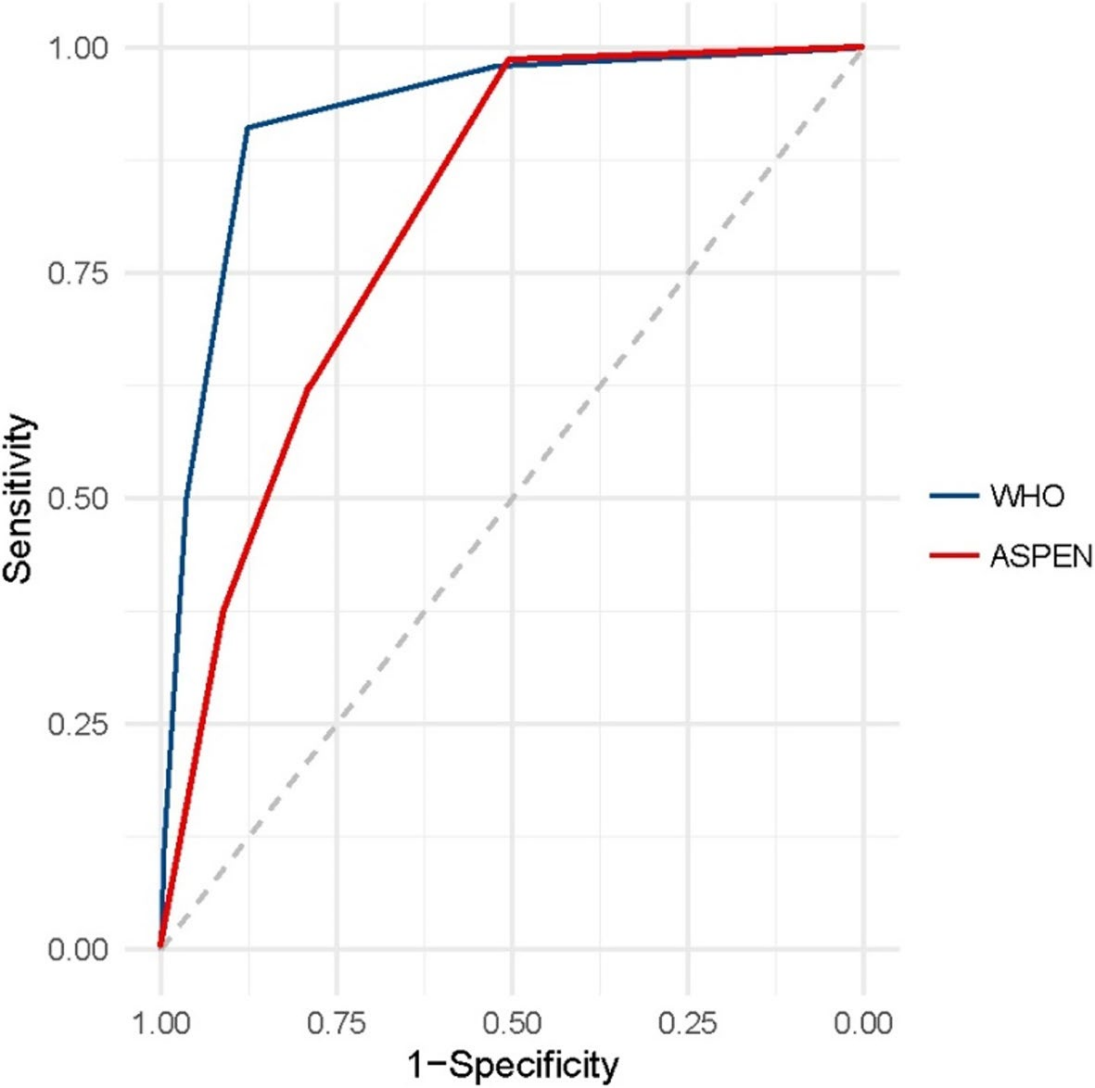
ROC curves of screening score using WHO and ASPEN standards. ROC, receiver operating characteristic; ASPEN, American Society for Parenteral and Enteral Nutrition

The ROC curve is used to judge the ability of risk identification and the closer the AUC (area under curve) is to 1, the better the identification effect. In our study, the AUC between malnutrition risk and non-risk was 0.924 (*P* < 0.01, 95% CI = 0.907–0.941) (Fig. 1). A critical value of 2 provided the best cut-off point (Youden index = 79.1%), with a sensitivity of 91.2% and a specificity of 87.9% (Table 3); the value was 0–1 for the non-malnutrition risk group and 2–5 for the malnutrition-risk group.

**Table 3:** Validation of cut-off value for screening score

Cut-off value	Sensitivity (%)	Specificity (%)	Youden index (%)
0.5	98.2	52.6	50.8
1.5	91.2	87.9	79.1
2.5	47.4	96.4	43.8
3.5	10.7	99.5	10.2
5.0	0.0	100.0	0.0

#### Screening results

When we assigned two points as the boundary, there were 351 cases in the risk group (accounting for 31.20% of the total) and 774 cases in the non-risk group (68.80%). Of these, 454 cases (40.36%) scored 0, 320 cases (28.44%) scored 1, 191 (16.98%) scored 2, 128 (11.38%) scored 3, and 33 (2.93%) scored 4. Two cases exhibited dietary-intake risk and 189 cases manifested disease risk.

#### Comparison of different reference standards

We adopted the WHO and ASPEN criteria for the screening results. According to the WHO standard, 272 cases were designated with malnutrition and the incidence rate was 24.18%; with 213 cases according to the ASPEN standard (18.93%). The values of sensitivity, specificity, positive-predictive value (PPV), and negative-predictive value (NPV) of each reference are shown in Table 5. The kappa test showed that the screening results were highly consistent with the WHO standard (κ = 0.720) and maintained a degree of consistency with the ASPEN standard (κ = 0.313) (Table 4). The comparison of ROC is shown in Fig. 1. None of the 61 WHO-standard overweight and obese cases were detected by the screening with nutritional risk.

**Table 4:** Comparison of WHO and ASPEN reference standards

Screening results	WHO	ASPEN
Sensitivity (%)	91.2	62.9
Specificity (%)	87.9	76.2
Positive-predictive value (PPV)%	70.7	38.2
Negative-predictive value (NPV)%	96.9	89.8
Cohen’s Kappa value (*p*)^a^	0.720 (< 0.01)	0.313 (< 0.01)

*ASPEN* American Society for Parenteral and Enteral Nutrition

^a^A kappa value > 0.6 represents a good level of agreement, < 0.2 is poor agreement

#### Discharge data

Screening data at discharge were available for 828 of the 1125 children (73.6%). Within this group, 82.00% of the children gained no weight or lost weight, and 18.00% gained weight. Forty-three patients (5.20%) had a weight loss of more than 5% during their admission. Children in the non-risk group had a significantly greater increase in weight between admission and discharge compared with the risk group (*p* < 0.001).

### Comparison of different nutritional risk groups

We found that the median age of the risk group was significantly less than that of the non-risk group (8.641 months [4.8, 23.1] vs. 31.589 months [12.4, 54.3], *P* < 0.01), and that the median length of hospital stay for the risk group (12.000 [8.0, 17.0]) was significantly longer than for the non-risk group (8.420 [5.0, 12.0], *P* < 0.01). The values for WFH Z, WFA Z, HFA Z, and BMI Z in the risk group were lower than those for the non-risk group (Table 5).

**Table 5:** Differences in clinical outcomes among nutritional-risk categories

	Risk group (*n* = 774)	Risk-free group (*n* = 351)	Statistical test
Age (months)	31.589 (12.4, 54.3)	8.641 (4.8, 23.1)	*p* < 0.01
WFH Z value	0.105 (− 0.5, 0.8)	− 0.730 (− 2.1, 0.8)	*p* < 0.01
WFA Z value	0.110 (− 0.6, 0.8)	− 1.840 (− 2.7, − 0.6)	*p* < 0.01
HFA Z value	− 0.080 (− 0.7, 0.7)	− 1.840 (− 2.8, − 0.7)	*p* < 0.01
BMI Z value	− 0.300 (− 1.1, 0.6)	− 1.730 (− 2.6, 0.6)	*p* < 0.01
Length of stay (days)	8.420 (5.0, 12.0)	12.000 (8.0, 17.0)	*p* < 0.01

*WFH Z* Weight for height Z value, *WFA Z* Weight for age Z value, *HFA Z* Height for age Z value

### Comparison of different age groups

There were significant differences in malnutrition risk among different age groups (χ^2^ = ? 144.933, *P* < 0.01). Children under one year of age exhibited the highest risk of malnutrition and longer hospitalization time (H = 78.085, *P* < 0.01). Children over five years had the shortest hospitalization time (Table 6).

**Table 6:** Risk differences by age group

	Age group				Statistical test
	0–	1–	3–	> = 5	
Risk group (cases [%])					
None	187 (46.98)	241 (75.31)	176 (86.70)	170 (83.33)	χ^2^ = 144.933, *p* < 0.01
Yes	211 (53.02)	79 (24.69)	27 (13.30)	34 (16.67)	
Length of stay (days)	11.390 (8.0, 16.0)	9.000 (5.5, 12.0)	7.580 (5.0, 11.7)	7.000 (5.0, 10.5)	*H* = 78.085, *p* < 0.01

### Comparison of different types of congenital heart disease

CHD is divided into cyanotic (associated with several anatomic abnormalities such as transposition of the great vessels, tetralogy of Fallot, etc.) and non-cyanotic. The non-cyanotic type can be subdivided into obstructive malformations (pulmonary artery stenosis, aortic coarctation, right heart position, etc.) and left-to-right shunt malformation (patent ductus arteriosus, ventricular septal defect, atrial septal defect, etc.) [28]. The risk of malnutrition was higher in those with cyanotic heart disease than in those with non-cyanotic heart disease (χ^2^ = 97.286, *p* < 0.01). Based on Bethesda classification, the cases were also divided into simple, moderate and complex groups. The results show significantly lower malnutrition risk in simple group, while higher risks in moderate and complex groups (χ^2^ = 400.694, *p* < 0.01) (Table 7).

**Table 7:** Risk differences by types of congenital heart disease (cases [%])

Classification	Risk group	Risk-free group	Statistical test
Cyanotic (*n* = 206)	118 (61.14)	75 (38.86)	χ^2^ = 97.286 *p* < 0.01
Non-cyanotic (*n* = 973)	233 (25.00)	699 (75.00)	
Simple (*n* = 535)	13 (2.43)	522 (97.57)	*χ*^*2*^ = *400.694* *p* < *0.01*
Moderate (*n* = 390)	209 (53.59)	181 (46.41)	
Complex (*n* = 200)	129 (64.50)	71 (35.50)	

### Influencing factor analysis of malnutrition at discharge

A binary logit regression analysis was performed for CHD children at discharge with possible influencing factors including gender, age, length of stay, disease risk and diet as independent variables and WHO criteria for malnutrition as dependent variables. As shown in Table 8, LOS and disease risk had a significant positive effect on malnutrition; age of month had a significant negative effect on malnutrition; but gender and diet did not have an effect on malnutrition.

**Table 8:** Analysis of influencing factors of malnutrition risk at discharge

Variables	Coefficient	SE	Wald χ^2^	p	OR	95% CI
Sex	0.284	0.194	2.140	0.143	1.328	0.908–1.943
Age (months)	− 0.020	0.004	22.593	0.000	0.980	0.972–0.988
Length of stay (days)	0.056	0.012	20.487	0.000	1.057	1.032–1.083
Disease risk	0.531	0.235	5.081	0.024	1.700	1.072–2.697

## Discussion

Children with CHD are often at greater risk for malnutrition and growth defects, as augmented sympathetic activity, elevated respiratory function, and congestive heart failure increase metabolic demand [5]. A higher incidence of gastrointestinal structural abnormalities, gastroesophageal reflux, and food intolerance also occur. For example, the rate of neonatal necrotizing enterocolitis (NEC) in children with CHD is 10–100 times higher than that in normal children. Furthermore, preoperative fasting may lead to undernutrition, which increases the risk for malnutrition [1].

The European Society for Pediatric Gastroenterology and Hepatology called for establishing a Nutrition Support Team (NST) that was focused on scientific and effective nutrition management of hospitalized children and a reduction in the prevalence of malnutrition [29]. Perioperative nutritional management is the key to improving the clinical prognosis of children with CHD. According to an expert consensus on nutritional support for children with CHD, the nutritional-screening evaluation suggests that screening be conducted within 24 h after admission, 3–7 days after surgery and before discharge, and once per week if the hospitalization time is over two weeks [30]. As a first step, nutritional risk screening requires convenient and practical tools. An ideal malnutrition-risk screening tool should be sensitive, accurate, and be easy to apply [7].

The earliest study of nutritional status of CHD children in China found incidence rates for acute and chronic malnutrition of 48.4% and 37.5%, respectively [31]. In our analysis, the incidence of malnutrition in children with CHD was 31.20%, which was similar to the data from several small-sample studies that used the Z-score method (30.30%–57.14%) [32, 33]. Other reports also validated the Canadian Infant Feeding & Nutrition Checklist for Congenital Heart Disease (IFNC: CHD) assessment tool [9]. However, none of the above tools were verified by multi-center and large-sample investigations with ideal sensitivity and specificity.

The factors that promote malnutrition in children with CHD are many and can include feeding and maternal behavior. In the present study, we used a modified pediatric malnutrition risk tool to screen the nutritional risk for children with CHD in China. Our data suggested that the risk of malnutrition in young children with cyanotic CHD was higher and the hospitalization time longer than in their counterparts. A limitation of this study is that the screening tool was employed by different nurses, possibly influencing the results whereas we provided a training session to all participants at the beginning of the project. Also, for the dietary intake part of the tool, different scores are assigned as “Normal, no obvious change”, “Less than or equal to 50% less than usual in the previous month”, and “Over the previous month, eating decreased by more than 50% compared with normal” from 0,1 to 2 respectively, while only two cases exhibited dietary intake risk. Such result might show that the intake measure does not strengthen the tool that it did not really depict the feeding situations of the children. For further studies, it is suggested that the feeding status, growth rate, CHD type and complications, postoperative complications, postoperative mechanical ventilation time, medication use, differentiation of corrected vs. non-corrected CHD in older children, and readmission rate be incorporated into the tool for analysis and further validation. A decrease in weight for age during the first months after surgery for congenital heart problems is strongly related to late mortality in children [34]. Hence, timely screening with a convenient tool such as the one in this study, nutritional support, and follow-up post-surgery should be conducted to improve clinical outcome to optimize the health of CHD children.

## Conclusion

The new digital pediatric malnutrition screening tool showed high sensitivity and specificity in children with CHD. It is convenient and easy to apply. The tool indicated that the malnutrition risk for young children and children with cyanotic or Bethesda moderate and complex CHD was higher, and the hospitalization time was longer than in the non-risk group. It also provides a rational approach to targeted nutrition intervention and support and may improve clinical outcomes.

## Data Availability

The datasets used and/or analyzed during the current study are available from the corresponding author on reasonable request.
